# Women's decision-making autonomy and its relationship with child feeding practices and postnatal growth

**DOI:** 10.1017/jns.2020.30

**Published:** 2020-08-27

**Authors:** Mahama Saaka

**Affiliations:** School of Allied Health Sciences, University for Development Studies, P.O. Box TL 1883, Tamale, Ghana

**Keywords:** Women's autonomy, Health care decision-making, Dietary diversity, Child growth outcomes, Ghana

## Abstract

Childhood stunting remains a global public health concern. Little has been documented on the effect of women's decision-making autonomy on child growth in settings where decision-making at the household and community levels is largely dominated by men. To assess the relationship between maternal autonomy and child growth, we analysed data from a cross-sectional study of 422 mothers and their youngest child aged 6–24 months in the Bawku West District of Ghana. The dimensions of women's autonomy measured were decision-making power, freedom of mobility and financial autonomy. We then compared how each dimension was associated with the likelihood of stunting and wasting. The important predictors of child growth and dietary intake as measured by the mean length-for-age *Z*-score (LAZ) and minimum acceptable (MAD) diet, respectively, were determined using multivariable regression models. The overall composite index of women autonomy (CIWA) showed that 52⋅8 % of women were of high autonomy and half of them had higher autonomy regarding their own and their children's health. After adjusting (multiple regression analysis) for potential confounders, the mean LAZ of children born to women of high autonomy was significantly higher than LAZ of children born to women of low autonomy (β = 0⋅132; 95 % CI 0⋅19, 0⋅95; *P* = 0⋅004). Similarly, high women's autonomy was a significant independent predictor of meeting MAD (AOR = 1⋅59; CI 1⋅09, 2⋅34). Of all, the dimensions of women's autonomy measured in this study, health care autonomy better predicted child growth and dietary intake. Based on the findings, nutrition policies and interventions that enhance women's decision-making autonomy could have a positive impact on child growth outcomes.

## Introduction

Childhood stunting (height-for-age *Z*-scores below −2) remains a global public health concern. It is well documented that malnutrition delays growth and development and also exposes individuals to increased risk of acquiring an infectious disease^(^^1)^ especially in developing countries, where malnutrition contributes to over 50 % of childhood deaths^(^^2)^. The problem of child malnutrition is multifaceted and one potential area that holds promise in the search for solutions to this is the enhancement of decision-making autonomy of the woman. Women's autonomy has been variously defined and measured. As in similar other studies, autonomy is defined as the ability to make decisions on one's own, to control one's own body and to determine how resources will be used, without needing to consult with or ask permission from another person^(3)^. Defined this way, women's autonomy denotes control, which is likely an important factor that influences childcare, and may improve nutritional outcomes^(4,5)^. A better understanding of how women's autonomy impacts on child nutritional status will hopefully project it as a vital public health intervention in developing nations to facilitate progress towards accomplishing many of the Sustainable Development Goals (SDGs).

Women in every society contribute significantly to the upkeep of family members but are often constrained in some societies in taking decisions that affect their health. It has been reported that more than half of the women in Africa, Asia, Latin America and the Caribbean have no say in everyday household decisions, like making large household purchases for daily need, visit to family or relatives on decisions regarding their health care^(6)^. A woman's degree of autonomy in the household may have an impact on her ability to make decisions in the best interest of children's health or may limit her ability to direct household resources to her children. It has also been reported that low empowerment status curtails women's ability to access health care^(7)^ and that women who have lower autonomy within their household suffer from undernutrition themselves^(8,9)^.

Emerging available evidence suggests a positive effect of women's autonomy on child nutritional outcomes in a number of settings, including Pakistan, Bangladesh, India and Tanzania^(4,10–12)^. However, some other studies conducted elsewhere including Kenya and Nepal showed that maternal autonomy has a limited or no influence on child nutrition measures^(13–16)^. The literature is, therefore, inconclusive on the role of maternal autonomy and its relationship with nutrition being complex^(4,17)^.

From the foregoing, it appears the role of women's autonomy in influencing childcare and nutritional outcomes may be context-specific and its relevance in some societies warrants further investigation.

To effectively fight malnutrition, there is an urgent need to identify all the key potential determinants. Though a lot of studies on the relation between women's autonomy on child nutritional status have been conducted, there still remain unanswered questions. For example, the number of studies measuring separate dimensions of autonomy in one study is fairly limited making it difficult to infer any trends^(4)^. Therefore, more research is needed to clarify whether different dimensions do have differing impacts on child nutrition as this will pave the way to focus on relevant public health interventions that will enhance the most important aspects of women's autonomy. Furthermore, the role of the child's age on the effect of women's autonomy on child nutritional status is also less investigated. Could child's age interact or moderate the effect of women's autonomy on child nutrition?

Furthermore, a few of the past studies on this subject matter have measured all three World Health Organization (WHO) child growth outcomes (weight-for-age, weight-for-height and height-for-age). Measuring all three nutritional outcomes would improve comparability among studies and ensure that important associations of autonomy on child nutritional status are not missed^(4)^.

This present study sought to provide answers to these knowledge gaps in this analytical cross-sectional study conducted in the Bawku West District of Ghana, where malnutrition is high and decision-making at the household and community levels is largely dominated by men. Women's autonomy in the control of resources is an essential ingredient for adequate health care. We, therefore, hypothesized that women who are more autonomous would be able to allocate time and resources towards her own and children's well-being.

## Methods

### Study setting

The study was conducted in the Bawku West District which is one of the thirteen districts and municipalities in the Upper East Region of Ghana. The district was created in 1988 under the local government system by Legislative Instrument (LI) 1442. The district covers an area of approximately 1070 km^2^, which constitutes about 12 % of the total land area of the Upper East Region.

The population of the Bawku West District, according to the 2010 Population and Housing Census, is 94,034 representing 9⋅0 % of the region's total population. Males constitute 48 % and females represent 52 % of the district's population. About ninety (90⋅8 %) percentage of the population is rural.

The main economic activity of the people is agriculture (74⋅2 %) involving crop production, livestock and fish farming. Some of the crops cultivated are maize, sorghum, millet, groundnut, cowpea, soybeans, yam, rice as well as cassava. The major animals reared include cattle, sheep, goats, fowls, guinea fowls and birds are practically reared in every home.

### Study design, population and sampling procedure

This study used a community-based analytical cross-sectional design. Using the formula for estimating sample size of single proportions with a 95 % confidence level (CI) and an assumed regional prevalence of 50 % of women that are autonomous and a margin of error of 0⋅05, making 10 % provision for dropouts in the study, the sample size was calculated to be 422.

Mothers/primary caregivers and their children under 2 years participated in the study. Mother–child pairs from each household were eligible for inclusion in the study. We used a two-stage sampling procedure which was done by first using simple random sampling to select 25 communities from a list of communities from four sub-sections across the Zebilla District. The primary sampling units in the selected clusters were households. The minimum sample required for each cluster was sixteen households which were selected using a systematic sampling technique. In households with more than one eligible child, one was chosen at random to participate in the study.

### Data collection

Quantitative data were collected using structured questionnaire through face-to-face interviews during house-to-house visits. The questionnaire comprised socio-demographic characteristics of mothers/caregivers, autonomy level of mothers and anthropometric measurements of mother and child.

#### Independent and dependent variables and their measurement

The main explanatory variable in this study was women's autonomy. There were two main outcome variables. The first was postnatal growth which was defined quantitatively as length-for-age *Z*-score (LAZ) and weight-for-length *Z*-score (WLH). The categorical equivalents of these as binary variables are stunted/not stunted and wasted/not wasted, respectively. A child was classified as stunted if HAZ is below −2 sd below the population median, wasted if WHZ is below −2 sd below the population median and underweight as weight-for-age below −2 sd from the median of the WHO Child Growth Standards^(18)^.

The other outcome variable was child dietary intake which was measured qualitatively in terms proportion of children aged 6–23 months who received minimum acceptable dietary intake and postnatal growth of children aged 6–24 months.

Other confounders included (1) age and gender of the child; (2) maternal education, and utilisation of prenatal care and (3) household wealth status and maternal age. The main independent and dependent variables were measured as follows.

#### Measurement of women's autonomy

Of the several dimensions of women's autonomy described in the literature, four were assessed in this study: health care autonomy, general maternal household decision-making autonomy, movement autonomy and financial autonomy.

The index for general decision-making autonomy was composed of five questions, including the following:

1. Who makes the decision if you need to buy clothes for yourself?
2. Who makes the decision if you need to buy large household items/furniture?
3. Who makes the decision when your children have stationeries/school needs to be addressed?
4. Who makes the decision on how to spend the family's income?
5. Who in your household usually has the final say on having another child?

The responses to the questions were scored as follows: two points for decisions made by the woman; one point by decisions made jointly by both the woman and her husband; zero for all of the decisions taken by others.

Health care autonomy was defined as women's autonomy over her own or child's health care and was measured using three questions as follows:

1. Who makes the decision whether a child is sick enough to go for treatment?
2. Who in your household usually has the final say on your own health care?
3. If you are ill and need to see a doctor, do you first have to ask someone's permission?

Financial autonomy was related to evidence of women's control over financial resources. It was composed of three items: Whether a woman could spend her earn money without consulting anyone?; Who decides how family income is spent?; Who makes the decision whether a woman should work outside of the home?

The mobility/social autonomy dimension measured a mother's freedom of movement through her ability to independently travel to various places, attend social events or visit family and friends. The index of freedom of movement consisted of five items pertaining to whether women are usually allowed to go to some places on her own: just outside her house or compound, local market to buy things, local health centre or doctor, neighbourhood for recreation and home of relatives or friends in the neighbourhood^(19)^. The responses were scored as 1 (no permission required) and 0 (yes permission always required).

An overall composite index of women autonomy (CIWA), combining the four dimensions was also calculated. Two categories (i.e. low and high) of the individual components and the CIWA were created on the basis of the average value of each variable in the study sample. The women scoring less than the average score were put in the low autonomy category and women of at least the average score were categorised as high autonomy.

### Anthropometric assessment

Anthropometric measurements of length/height and weight were taken by two people, one who was primarily responsible for positioning the child and reading the measurement and another who made sure the position was correct and recorded the measurement. All measurements were recorded to one decimal place.

The length measurement was taken with the child lying correctly in an infantometer with the movable end as a foot piece. Length measurements were taken for children below 2 years who could not stand properly for a correct height measurement. Where the length was measured for children above 2 years, 0⋅7 cm was subtracted from the measurement to convert it to height. Height measurement was taken for children above 2 years using an infantometer and mothers using a microtoise. Height was measured when subjects were standing straight with feet together, buttocks and lower back touching the wall (infantometer), subject looking straight ahead without footwear and long hair well-positioned. Weight measurement was taken for mothers and children on the scale with minimal movements of subjects on the scale and minimal clothing.

We assessed nutritional status outcomes including length-for-age (LAZ), weight-for-age (WAZ) and weight-for-length or (WLZ) calculated using the WHO 2005 growth standards^(18)^.

#### Age determination

Dates of birth for children in this study were taken from their health records booklets and other relevant sources available. Mothers’ ages were taken from documents such as National Voter's ID cards, Health Insurance ID cards, Mother's Pregnancy Antenatal Booklets and estimated age for others based on the age of their firstborn and other locally important calendar events such as the 1982 famine in Ghana.

##### Measurement of infant and young child feeding (IYCF) practices

One round of quantitative 24-hour dietary recall for the previous day was used to estimate the dietary practices of children^(20)^. The specific IYCF indicators assessed were timely initiation of breast feeding, timely complementary feeding, minimum dietary diversity, minimum meal frequency and minimum acceptable diet (MAD), appropriate complementary feeding and Infant and child feeding index (ICFI), a composite index which measures complete feeding practices for infants and young children.

The overall ICFI computed comprised feeding practices, including currently breast-feeding status, timely initiation of breast feeding within 1 hour, avoidance of prelacteal feeding, avoidance of bottle feeding, introduction of complementary foods at 6 months, minimum meal frequency, minimum dietary diversity and feeding child with colostrum.

Minimum dietary diversity is defined by the WHO as the proportion of children who received foods from at least four out of seven food groups. Minimum meal frequency was defined as children 6–36 months of age who received solid, semi-solid or soft foods a minimum number of times in the previous day. The minimum required frequency varied by child age and breast-feeding status (6–8 months is two times, 9–11 months is three times and 12–23 months is three times). MAD is a combined indicator of both minimum dietary diversity and minimum meal frequency.

The seven foods groups used for the assessment of IYCF practices were (1) grains, roots and tubers; (2) legumes and nuts; (3) dairy products; (4) flesh foods; (5) eggs; (6) vitamin A-rich fruits and vegetables and (7) other fruits and vegetables.

##### Assessment of socio-economic status

A household wealth index based on household assets and housing quality was used as a proxy indicator for socio-economic status (SES) of households. Principal Component Analysis (PCA) was used to determine household wealth index from information collected on housing quality (floor, walls and roof material), source of drinking water, type of toilet facility, the presence of electricity, type of cooking fuel, and ownership of modern household durable goods and livestock (e.g. bicycle, television, radio, motorcycle, sewing machine, telephone, cars, refrigerator, mattress, bed, computer and mobile phone)^(21–24)^. The first principal component which explains as much of the variability in wealth index as possible was used to classify respondents with respect to SES. The different assumptions of PCA that data must meet for PCA to give valid results were as follows:

1. *The variables* were measured at the *continuous or ordinal levels*.
2. The variables were closely related and so there was some amount of *linear relationship between the variables*. The reason for this assumption is that a PCA is based on Pearson correlation coefficients, and as such, there needs to be a linear relationship between the variables.
3. There was *sampling adequacy*, which simply means that for PCA to produce a reliable result, large enough sample sizes are required. The 422 sample size used was more than the recommended minimum sample size of 150.

### Statistical data analysis

The quantitative data were analysed using IBM SPSS statistic version 22 (SPSS Inc., Chicago, Illinois). WHO Anthro software version 3.2.2 was used to convert anthropometric measurements to *Z*-scores which were then transferred to SPSS software for further analyses. Anthropometric *Z*-scores of height-for-age (HAZ) and weight-for-height (WHZ) were determined. Before testing for associations between independent and the dependent variables, the data were cleaned. The raw data were checked for accuracy and consistency according to a standard procedure including double entry and running a frequency distribution on each of the variables in SPSS software. The main aim was to look for data entry errors and patterns to determine the primary sources of data inaccuracy. For example, if a variable with a Likert scale ranging from 1 to 5, all of your values should be in this range. The anthropometric data were checked for values outside the expected ranges. *Z*-scores which were outside the WHO flags: WHZ −5 to 5; HAZ −6 to 6 and WAZ −6 to 5 were excluded from the data set.

Bivariable and multivariable analyses were performed to identify the determinants of stunting, overweight and wasting. Bivariable analyses were performed using Chi-square (*χ*^2^) tests and some cases Fisher's Exact Test. All explanatory variables that showed statistically significant association in the bivariable analysis with the outcome variables were entered to a multivariable linear regression model to identify independent factors associated with length-for-age *Z*-scores. Before running the regression, Pearson correlation analysis was run to eliminate highly correlated independent variables that had a correlation coefficient greater than 0⋅70^(25)^.

The important predictors of stunting and MAD were determined using binary logistic regression. Odds ratios (ORs) along with CIs were used to determine the association between predictor variables and outcome measures, considering significant association at α <0⋅05.

## Ethical considerations

The study protocol was approved by the School of Allied Health Sciences, University for Development Studies. Informed consent was also obtained from study participants after needed information and explanation. In situations, where the respondent could not write or read, verbal informed consent was sought from all the study participants before the commencement of any interview.

### Results

#### Socio-demographic characteristics of the study sample

The mean (+sd) age of mothers was 31⋅83 (6⋅83) years with a range of 18–65 years. The mean (+sd) age of children was 14⋅17 (8⋅007) months. The sample comprised 55⋅9 % male children and the majority of the children were in the age group 12–23 months. Many of the respondents (49⋅5 %) had no formal education at all and 86⋅3 % were married. Farming (30⋅8 %) was the commonest occupation among the mothers (Table 1).

**Table 1.** Socio-demographic characteristics of the sample

Characteristics	Frequency (*n*)	%
Age of children (months)		
Under 12 months	151	35⋅8
12–23 months	208	49⋅3
24–36 months	63	14⋅9
Maternal age (years)		
Under 25 years	59	14⋅0
25–34 years	212	50⋅2
At least 35 years	151	35⋅8
Educational level of mothers		
None	209	49⋅5
Low (Primary and Junior High School (JHS))	127	30⋅1
High (at least Senior High School (SHS))	86	20⋅4
Household wealth index		
Low (<median of 8)	181	42⋅9
High (at least 8)	241	57⋅1
Religion		
Christianity	209	49⋅5
Islam	185	43⋅8
ATR	27	6⋅4
Occupation		
Trader	98	24⋅5
Farmer	123	30⋅8
Service provider	67	16⋅8
Salary worker (e.g. teacher and health worker)	10	2⋅4
Nothing	102	25⋅5
Ethnicity		
Kusasi	322	76⋅3
Mamprusi	12	2⋅8
Moshi	17	4⋅0
Others	71	16⋅9

#### Nutritional status of mothers and children aged 6–24 months

The prevalence of stunting, wasting and underweight in the study sample was 27⋅7, 7⋅8 and 9⋅2 %, respectively (Table 2). Out of 422 mothers, 4⋅5 % of them having heights less than 150 cm, while 6⋅9 % were less than 18⋅5 kg/m^2^.

**Table 2.** Nutritional status of mothers and children aged 0–36 months

Characteristics	Mean ± sd	Frequency (*n*)	%
Nutritional status			
Height-for-age *Z*-score	−0⋅92 ± 2⋅2		
Weight-for-height *Z*-score	0⋅28 ± 1⋅7		
Weight-for-age *Z*-score	−0⋅38 ± 1⋅33		
% Stunted (HAZ < −2)		117	27⋅7
% Wasted (WHZ < −2)		33	7⋅8
% Underweight (WAZ < −2)		39	9⋅2
Maternal short stature <150 cm (%)		19	4⋅5
Maternal BMI<18⋅5 kg/m^2^		29	6⋅9

The mean *Z*-scores indicate that the infants in our sample were much shorter and lighter, compared to the international standard.

#### Levels of maternal autonomy in the study population

Table 3 shows the aspects of maternal decision-making autonomy, while Table 4 shows the level of freedom of movement. In this study, 82⋅9 % of women were autonomous in what to do when the child fell sick facilities, while 85⋅1 % of women were involved in making household purchases for daily needs. Decisions regarding all movements outside the house to some extent were restricted (i.e. warrants seeking permission from either husband or a senior household member).

**Table 3.** Maternal involvement in household final say on key decisions

Decision	Frequency (*n*)	%
Your own health care?	366	86⋅7
Making large household purchases?	292	69⋅2
Making household purchases for daily needs?	359	85⋅1
Visits to family or relatives?	298	70⋅6
What food should be cooked each day?	327	77⋅5
You should do work to earn money?	342	81⋅0
What to do if a child falls sick?	350	82⋅9
Having another child?	282	66⋅8

**Table 4.** Are you usually allowed to go to the following places on your own?

Place	Frequency (*n*)	%
Freedom to see a doctor when sick	178	42⋅2
Just outside your house or compound?	394	93⋅4
Local market to buy things?	418	99⋅1
Local health centre or doctor?	405	96⋅0
In the neighbourhood for recreation?	341	80⋅8
Home of relatives or friends in the neighbourhood?	308	73⋅0

The overall CIWA was categorised into low and high. Accordingly, 52⋅8 % of the respondents were of high autonomy and general maternal decision-making autonomy was the most restricted. The overall level of women's autonomy, as well as its components, is shown in Table 5.

**Table 5.** Dimensions of women's autonomy

Characteristics	Frequency (*n*)	%
Composite index of women autonomy (CIWA)		
Low	199	47⋅2
High	223	52⋅8
Health care autonomy		
Low	211	50⋅0
High	211	50⋅0
General maternal decision-making power		
Low	294	69⋅7
High	128	30⋅3
Maternal financial independence		
Low	251	59⋅5
High	171	40⋅5
Freedom of movement		
Low	108	25⋅6
High	314	74⋅4

#### Prevalence of child malnutrition among women with different levels of autonomy

The prevalence of stunting and wasting as stratified by maternal autonomy is shown in Table 6. Health care autonomy, general maternal decision-making power and CIWA were negatively associated with stunting, but no discernible association existed between overall/components of maternal autonomy and wasting. Maternal freedom of movement (freedom of movement to homes of relatives) was associated negatively with stunting. Maternal financial independence was not associated with stunting.

**Table 6.** Prevalence of child malnutrition among women with different levels of maternal autonomy

	% Stunted	Test statistic	% Wasted	Test statistic
Composite index of women autonomy (CIWA)				
Low (<mean score of 10)	34⋅2	*χ*^2^ = 7⋅8; *P* = 0⋅005	7⋅0	*χ*^2^ = 0⋅3; *P* = 0⋅6
High (at least 10)	22⋅0		8⋅5	
Health care autonomy				
Low (< mean score of 3)	32⋅2	*χ*^2^ = 4⋅3; *P* = 0⋅04	8⋅1	*χ*^2^ = 0⋅03; *P* = 0⋅9
High (at least 3)	23⋅2		7⋅6	
General maternal decision-making power				
Low (< mean score of 5)	31⋅6	*χ*^2^ = 7⋅39; *P* = 0⋅007	7⋅1	*χ*^2^ = 0⋅6; *P* = 0⋅4
High (at least 5)	18⋅8		9⋅4	
Maternal financial independence				
Low (< mean score of 3)	29⋅5	*χ*^2^ = 0⋅9; *P* = 0⋅3	7⋅6	*χ*^2^ = 0⋅1; *P* = 0⋅8
High (at least 3)	25⋅1		8⋅2	
Freedom of movement to homes of relatives				
No	35⋅1	*χ*^2^ = 4⋅2; *P* = 0⋅04	10⋅5	*χ*^2^ = 1⋅6; *P* = 0⋅2
Yes	25⋅0		6⋅8	

#### Comparison of child growth and dietary indicators according to maternal health care autonomy

Table 7 shows unadjusted differences in mean child growth indices according to maternal health care autonomy using analysis of variance (ANOVA). The unadjusted values of mean length-for-age *Z*-score (LAZ) and child dietary diversity were positively associated with high *women's autonomy*, but weight-for-age (WAZ) and weight-for-length (WLZ) were not associated.

**Table 7.** Comparison of child growth and dietary indices according to maternal health care autonomy (Unadjusted differences)

Indicator	Women's autonomy	*N*	Mean	sd	95 % confidence interval for Mean		Test statistics
					Lower bound	Upper bound	
HAZ	Low	211	−1⋅16	2⋅17	−1⋅46	−0⋅87	*F* (1, 421) = 5⋅65; *P* = 0⋅02
	High	211	−0⋅67	2⋅12	−0⋅96	−0⋅38	
WHZ	Low	211	0⋅27	1⋅76	0⋅029	0⋅51	*F* (1, 421) = 0⋅02; *P* = 0⋅9
	High	211	0⋅29	1⋅70	0⋅06	0⋅52	
WAZ	Low	211	−0⋅50	1⋅23	−0⋅66	−0⋅33	*F* (1, 421) = 3⋅49; *P* = 0⋅06
	High	211	−0⋅27	1⋅24	−0⋅4394	−0⋅10	
Child dietary diversity score (DDS)	Low	211	2⋅44	1⋅78	2⋅20	2⋅68	*F* (1, 420) = 6⋅54; *P* = 0⋅01
	High	210	2⋅89	1⋅83	2⋅64	3⋅14	
Maternal DDS	Low	211	4⋅68	1⋅53	4⋅47	4⋅89	*F* (1, 421) = 1⋅97; *P* = 0⋅2
	High	211	4⋅48	1⋅38	4⋅29	4⋅67	
BMI of mother	Low	211	23⋅35	6⋅30	22⋅49	24⋅20	*F* (1, 421) = 0⋅09; *P* = 0⋅8
	High	211	23⋅19	3⋅43	22⋅73	23⋅66	
Maternal weight	Low	211	62⋅00	10⋅53	60⋅57	63⋅43	*F* (1, 421) = 1⋅9; *P* = 0⋅2
	High	210	65⋅62	36⋅03	60⋅72	70⋅52	

Using analysis of covariance (ANCOVA) that adjusted for age of the child, height of mother, gender of the child, WLZ, age of mother and religion of mother, there was a significant difference in mean LAZ between children of mothers who had high health care autonomy and children's whose mothers had low health care autonomy (−0⋅674 *v*. −1⋅159; 95 % CI 0⋅02, 0⋅65; *P* = 0⋅04).

#### Determinants of mean length-for-age *Z*-scores

A number of explanatory variables were tested for their association with LAZ. The variables tested included women's autonomy and its components, age and sex of child, current breast-feeding status, diarrhoeal infection, utilisation of antenatal care services, birth interval, parity, maternal height, maternal educational level, household wealth index, source of drinking water, type of toilet facility, bottle feeding, minimum dietary diversity score and number of under five children in household.

After adjusting (multiple regression analysis) for maternal and child characteristics confounders, women's autonomy was significantly and positively associated with length-for-age *Z*-score (LAZ). The mean LAZ of children born to women of high autonomy was significantly higher than LAZ of children born to women of low autonomy (β = 0⋅132; *P* = 0⋅004; Table 8).

**Table 8.** Determinants of mean length-for-age *Z*-scores (Multivariable regression analysis)

Model	Standardized coefficients	Sig.	95 % confidence interval for β		Collinearity statistics	
	β		Lower bound	Upper bound	Tolerance	Variance inflation factor (VIF)
(Constant)		0⋅000	−15⋅32	−6⋅94		
BMI of mother	0⋅118	0⋅016	0⋅011	0⋅11	0⋅834	1⋅199
High women's autonomy	0⋅132	0⋅004	0⋅19	0⋅95	0⋅988	1⋅012
Child wasting	0⋅304	<0⋅001	1⋅71	3⋅12	0⋅989	1⋅012
Height of mother	0⋅175	<0⋅001	0⋅02	0⋅06	0⋅820	1⋅219
African Traditional Religion (ATR)	0⋅144	0⋅001	0⋅20	0⋅81	0⋅985	1⋅016
Female child	0⋅115	0⋅011	0⋅115	0⋅88	0⋅991	1⋅010
No bottle feeding	0⋅111	0⋅013	0⋅13	1⋅08	0⋅990	1⋅010

Female children were generally taller than male children by 0⋅115 standard units (β = 0⋅115; *P* = 0⋅01). A unit increase in the height of mother increases the mean LAZ increases by 0⋅175 standard units (*P* < 0⋅001). Low WHZ (i.e. wasting) was the prominent determinant of LAZ as wasted children were taller by 0⋅304 standard units (β = 0⋅304; *P* < 0⋅001). The mean LAZ of children born to mothers who follow African Traditional Religion (ATR) was significantly higher than LAZ of children born to Christian mothers. A unit increase in mother's body mass index (BMI) was associated with a 0⋅118 increase in LAZ (*P* = 0⋅016). Children who were not bottle-fed were taller than bottle-fed children by 0⋅111 standard units (β = 0⋅111; *P* = 0⋅01).

Similar determinants of postnatal child growth including women's autonomy were obtained when the outcome variable was treated as a categorical variable (i.e. stunted or not stunted). Logistic regression analysis showed that low autonomy, Christians, male child, low BMI of mother, bottle feeding, child not wasted, and height of mother were independently associated with an increased odd of stunted growth (Table 9). Compared to women of high autonomy, women of low autonomy were 1⋅9 times more likely of having stunted children (AOR = 1⋅93; CI 1⋅21, 3⋅07).

**Table 9.** Determinants of child stunting (Binary logistic regression analysis)

	Wald	Sig.	Exp(β)	95 % CI for Exp(β)	
				Lower	Upper
Low autonomy	7⋅606	0⋅006	1⋅927	1⋅209	3⋅071
Religion (Reference: African traditional religion (ATR))	6⋅631	0⋅04			
Christianity	4⋅983	0⋅03	4⋅25	1⋅19	15⋅17
Islam	2⋅561	0⋅110	2⋅863	0⋅79	10⋅38
Male child	5⋅696	0⋅02	1⋅78	1⋅11	2⋅86
BMI of mother	8⋅673	0⋅003	0⋅91	0⋅86	0⋅97
Bottle feeding	5⋅123	0⋅024	1⋅88	1⋅09	3⋅26
Not wasted	6⋅318	0⋅012	4⋅88	1⋅42	16⋅80
Height of mother	14⋅065	<0⋅001	0⋅95	0⋅92	0⋅98
Constant	5⋅114	0⋅024	437⋅51		

Women who were Christians were 4⋅3 times more likely (AOR = 4⋅25; CI 1⋅19, 15⋅17) of having stunted children, compared to women who were followers of ATR. Bottle-fed children who were 1⋅9 times (AOR  = 1⋅88; CI 1⋅09, 3⋅26) more likely to be stunted, compared to their counterparts who were not bottle-fed. Male children were 1⋅8 times more likely (AOR = 1⋅78; CI 1⋅11, 2⋅86) to be stunted, compared to female children.

Children who were not wasted were 4⋅9 times more likely (AOR = 4⋅88; CI 1⋅42, 16⋅80) to be stunted children, compared to children who were wasted.

A unit increase in maternal height was associated with 5 % protection against child stunting (AOR = 0⋅95; CI 0⋅92, 0⋅98). Similarly, a unit increase in a mother's BMI was associated with 9 % protection against child stunting (AOR = 0⋅91; CI 0⋅86, 0⋅97).

The set of variables alone accounted for 17⋅1 % (Nagelkerke R Square = 0⋅171) of the variability in stunted growth in children. It is also an indication that other factors contribute to the dependent variable but were not measured in this study.

An additional interaction term between the autonomy variable and age grouping of child was included in the logistic regression model to assess whether the age of children interacts with the relationship between women's autonomy and stunting. The interaction term was very significant after adjusting for some potential covariates (AOR = 0⋅71; CI 0⋅56, 0⋅90; *P* = 0⋅004). The strongest association between women's autonomy and stunting was found among children aged 12–23 months, compared to other groups of children.

As a result of the interaction between women's autonomy and age of child, the protective effect of autonomy varied across age groups. Whereas high autonomy protected 60 % of the probability of stunting among children aged 12–23 months, there was only an insignificant reduction in children aged 6–11 months. There was no discernible beneficial benefit of women's autonomy on stunting among children under 6 months and children aged 24–36 months (Table 10).

**Table 10.** Crude odds ratio (COR) of women's autonomy for stunting according to age of child

	Age groups of children (months)				
	Under 6	6–11	12–23	24–36	All ages
Child-level equation Intercept for stunting (constant)	−0⋅575	−0⋅804	−0⋅568	−0⋅754	−0⋅656
Standard error (se)	0⋅550	0⋅530	0⋅323	0⋅599	0⋅220
Wald	0⋅862	3⋅077	8⋅105	1⋅502	7⋅712
Odds ratio (Exp(β))	1⋅67	0⋅39	0⋅40	0⋅48	0⋅54
95 % confidence interval for Exp (β)	0⋅57, 4⋅90	0⋅14, 1⋅12	0⋅21, 0⋅75	0⋅15, 1⋅55	0⋅35, 0⋅84
*P* value	0⋅4	0⋅08	0⋅004	0⋅22	0⋅005

#### Relationship between women's autonomy and child feeding practices

After controlling for confounders, women with higher overall women's autonomy were more likely to feed their children with the minimum required dietary diversity (AOR = 1⋅69; CI 1⋅12, 2⋅54) as compared with women with lower autonomy. Similarly, higher women's autonomy was associated with a higher likelihood of feeding an MAD (AOR = 1⋅59; CI 1⋅09, 2⋅34). The only component that associated positively with MAD in this study population was maternal financial independence (Table 11). Overall women's autonomy, maternal financial independence and freedom of movement to homes of relatives were also associated with a composite feeding index which measured complete feeding practices for infants and young children.

**Table 11.** Association between women's autonomy dimensions and IYCF practices among children aged 6–23 months

Autonomy/Dimension	AOR (95% CI) of IYCF practices^b^		
	Minimum dietary diversity	Minimum acceptable diet	Classification of ICFI
Composite index of women autonomy (CIWA)	1⋅69 (1⋅12, 2⋅54)^a^	1⋅59 (1⋅09, 2⋅34)^a^	1⋅52 (1⋅04, 2⋅22)^a^
Health care autonomy	1⋅38 (0⋅89, 2⋅12)	1⋅52 (0⋅97, 2⋅36)	1⋅06 (0⋅66, 1⋅70)
General maternal decision-making power	1⋅34 (0⋅90, 1⋅99)	1⋅18 (0⋅81, 1⋅71)	1⋅31 (0⋅90, 1⋅91)
Maternal financial independence	1⋅52 (0⋅99, 2⋅32)	1⋅84 (1⋅23, 2⋅75)^a^	1⋅68 (1⋅11, 2⋅54)^a^
Freedom of movement to homes of relatives	1⋅46 (0⋅89, 2⋅40)	1⋅14 (0⋅68, 1⋅89)	1⋅90 (1⋅13, 3⋅20)^a^

a Significant.

b Confounders adjusted for include breast-feeding status, child age, mothers’ occupation type, timely introduction of complementary food at 6 months.

As presented in Table 12, there was a strong positive association between overall women's decision-making autonomy and key child feeding indicators including timely initiation of breast feeding (*P* < 0⋅001), feeeding of diversified diets (*P* = 0⋅001), MAD (*P* = 0⋅004) and the overall composite ICFI (*χ*^2^ = 5⋅9; *P* = 0⋅02). However, no association was observed between women's autonomy and timely introduction of complementary foods.

**Table 12.** Association between child feeding practices and overall women's autonomy (Bivariable analysis)

Characteristic	Classification of overall women's autonomy		Test statistic
	Low *n* (%)	High *n* (%)	
Timely initiation of breast feeding			
No	83 (40⋅5)	82 (25⋅9)	*χ*^2^ = 12⋅4; *P* < 0⋅001
Yes	122 (59⋅5)	235 (74⋅1)	
Minimum dietary diversity – children			
Less than 4	102 (49⋅8)	111 (35⋅0)	*χ*^2^ = 11. 1; *P* = 0⋅001
At least 4	103 (50⋅2)	206 (65⋅0)	
Timely introduction of complementary foods			
No	122 (59⋅5)	183 (57⋅7)	*χ*^2^ = 0⋅2; *P* = 0⋅7
Yes	83 (40⋅5)	134 (42⋅3)	
Minimum acceptable diet			
No	116 (56⋅6)	139 (43⋅8)	*χ*^2^ = 8⋅1; *P* = 0⋅004
Yes	89 (43⋅4)	178 (56⋅2)	
Appropriate complementary feeding rate			
No	166 (81⋅0)	226 (71⋅3)	*χ*^2^ = 6⋅2; *P* = 0⋅01
Yes	39 (19⋅0)	91 (28⋅7)	
Classification of Infant and child feeding index (ICFI)			
Low	77 (37⋅6)	87 (27⋅4)	*χ*^2^ = 5⋅9; *P* = 0⋅02
High	128 (62⋅4)	230 (72⋅6)	

#### Determinants of meeting MAD (Binary logistic regression analysis)

After controlling for potential confounding factors in logistic regression analysis, high women's autonomy remains a significant independent predictor of meeting MAD (AOR  =  1⋅59; CI 1⋅09, 2⋅34). Other determinants included were age of the child, children whose mothers were farmers, timely introduction of complementary foods at 6 months and non-breast-feeding children. Older children (12–23 months) were associated with 4⋅38 odds of meeting MAD (AOR  =  4⋅38; CI 2⋅86, 6⋅69). Children who were timely introduced to complementary foods at 6 months were 2⋅0 times more likely of meeting MAD (AOR  =  2. 08; CI 1⋅42, 3⋅07), compared with their colleagues who were fed with such foods on any other time. Children whose mothers reported they were farmers were 1⋅7 times more likely of meeting MAD (AOR  =  1. 69; CI 1⋅08, 2⋅65), compared to children whose mothers were non-farmers (Table 13). The set variables alone accounted for 20⋅5 % (Nagelkerke R Square = 0⋅205) of the variability in MAD.

**Table 13.** Determinants of meeting minimum acceptable diet (Binary logistic regression analysis)

	Wald	Sig.	Exp(β)	95 % CI for Exp(β)	
				Lower	Upper
High women autonomy	5⋅635	0⋅018	1⋅59	1⋅09	2⋅34
Occupation (Farmer)	5⋅251	0⋅022	1⋅69	1⋅08	2⋅65
Child's age (Reference: 6–11 months)	46⋅459	<0⋅001			
12–23 months	46⋅459	<0⋅001	4⋅38	2⋅86	6⋅69
24–35 months	5⋅437	0⋅020	2⋅361	1⋅147	4⋅861
Timely introduction of complementary foods	13⋅940	<0⋅001	2⋅084	1⋅417	3⋅065
Not currently breast feeding	3⋅788	0⋅052	2⋅055	0⋅995	4⋅244
Constant	41⋅836	0⋅000	0⋅156		

## Discussion

This present study sought to assess the relationship between women's autonomy, child feeding practices and growth indicators in a setting where malnutrition is high and decision-making at the household and community levels is largely dominated by men. The main finding was that overall maternal autonomy, positively associated with child feeding practices and protected against child stunting after controlling for key confounders such as gender of child, bottle feeding and height of mother.

The study findings further suggest that not all dimensions of women's autonomy were positively associated with child growth. The basic assumption in the study was that access to and control of resources is an essential ingredient for adequate health care and for that matter autonomous women may cater very well for their own health and that of their children. The health care autonomy decision-making was the best predictor of child growth, but maternal financial independence dimension associated more with child feeding practices. Women's autonomy which is a multidimensional, intangible and latent concept is expressed in several ways, such as decision-making power and control of household resources and the extent of freedom of movement^(26–28)^.

### Women's autonomy level in the study population

In our study sample, though presumed to be male-dominated, the majority of women had high autonomy with regards to decision-making on important matters that affect them. This finding collaborates that of Sethuraman *et al*.^(29)^, who reported that the majority of mothers make an important decision in their various household. Decisions regarding movements outside the house to some extent were generally free though some were restricted (i.e. warrants seeking permission from either husband or a senior household member). Similarly, other studies found out that the majority of the mothers could go to the health centres on their own without seeking permission from their husbands or a senior household member^(30)^.

### Association between dimensions of women's autonomy and child growth indicators

Though available evidence suggests a positive relationship between maternal autonomy and better child nutritional status^(4,31)^, little is known about how the various dimensions of women's autonomy influence postnatal child growth. The present study investigated the association between stunted growth and overall women's autonomy and its dimensions.

Health care autonomy, general maternal decision-making power and CIWA were negatively associated with stunting, but no discernible association existed between overall/dimensions of maternal autonomy and wasting. Maternal freedom of movement, particularly, freedom of movement to homes of relatives was associated negatively with stunting. This finding supports that of Shroff *et al*.^(5)^ who found out that not needing permission to go the market decrease the likelihood that a child will be stunted. However, maternal financial independence was not associated with stunting in our sample.

Similar findings have been reported that, when mothers have autonomy over child feeding and childcare, they may be more likely to follow recommended feeding practice and can reduce the likelihood of stunting among children^(32,33)^.

Similarly, after controlling for maternal and child characteristics confounders, the adjusted values of mean length-for-age *Z*-score (LAZ) were significantly and positively associated with health care autonomy decision-making power, but weight-for-age (WAZ) and weight-for-length (WLZ) were not.

As reported in other studies, the results of our study indicated that neither the overall women's autonomy nor its components had a significant effect on children's nutrition as measured by WLZ scores^(3,33)^ though others have reported of a positive association between mother's autonomy score and WLZ^(3)^. For example, in a Kenyan study, greater levels of women's autonomy were significantly associated with improved scores of WLZ scores among older children aged 3–10 years but not with children younger.

WLZ provides a recent measure of children's health compared to LAZ, and therefore, one would have expected that women's autonomy which, reflects the current ability of women to control household resources will negatively associate with child wasting. It is, however, important to note that prolong wasting can lead to stunting^(34,35)^, which is an outcome of chronic deprivation. Furthermore, though women's autonomy was measured as the current ability of women to make independent decisions regarding among others control of household resources, it is possible that this multidimensional characteristic of autonomy could remain stable through time and can impact on stunting.

The results of the present study show that the overall maternal autonomy had a significant positive impact on LAZ and a negative impact upon stunting. This finding confirms that of earlier studies carried out in many geographical locations^(33)^.

In other places, including rural Nepal, India and Haiti, higher health care decision-making power and mobility autonomy are reported to be significant predictors of improved height-for-age *Z*-scores after controlling for potential covariates^(3,11,30,33,36,37)^. This relationship may be partly explained by the fact that mothers with greater autonomy may benefit in other ways that indirectly affect their child. For example, they are more likely to make greater use of available health services such as antenatal care. Past studies have reported that women with greater autonomy are more likely to seek health care for themselves and use different forms of health care services available to them^(26,38,39)^. However, some other studies conducted elsewhere including Kenya and Nepal showed that maternal autonomy has a limited or no influence on child nutrition measures^(13–16)^. The discrepancies may be attributed to differences in outcome measures, ages of target children and the measurement of autonomy. For example, in the Kenyan study, the impact of women's autonomy on children's health was focused on short-term measurements of children's nutrition and growth, such as WHZ scores. Another possible reason for the conflicting results in these studies is how autonomy has been defined and measured by different researchers. In our study, we had to distinguish between general household decision-making from health-related decision-making autonomy. In the measurement of autonomy, the Kenyan study lumped general and health-related decision-making questions together and some of these may not be sensitive to child growth determinants. There is also the issue of some studies combining different components into a composite index and treating that as overall autonomy.

A possible interaction between age of child and women's autonomy on stunting was also investigated and found to be significant. The protective effect of autonomy against stunting varied according to age group of the child and the highest protection of 60 % was found among children aged 12–23 months. There was, however, no discernible beneficial benefit of women's autonomy on stunting among children under 6 months and children aged 24–36 months (Table 10). The children under 6 months of age will mostly be on breastmilk and may, therefore, not be sensitive to the benefits of women's autonomy.

Available evidence strongly suggests that the first 2 years of life are considered to be the most important ‘window of opportunity’ to make a long-term impact upon children's nutritional status^(40)^. Therefore, the finding that more autonomous mothers are able to protect against stunted growth especially during a specific period of the window is very crucial for policy and programming purposes.

### Mechanism of the relationship between women's autonomy and child nutrition

Based on the findings of the present study and others, an important question that has not been fully answered is the how and why greater autonomy leads to better child nutrition outcomes: What kind of decisions do women of greater autonomy make that improve the nutritional outcomes of their children?

Emerging evidence suggests that the positive link between women's autonomy and child nutrition may be explained by care behaviours shown by mothers. It has been shown that care behaviours are crucial for children's optimal growth, development and survival^(41)^. Availability of resources to caregivers is crucial in determining positive care behaviours. Among others, women's autonomy determines how well positive care behaviours will be exhibited. Autonomy indicates the ability of caregivers to have a control of their surroundings^(4,41)^. In many societies around the world, mothers are primary caregivers, and resources available to them may play a pivotal role in determining care behaviours^(41,42)^. Care behaviours comprise infant and young child feeding (IYCF), hygiene, health-seeking, and family care and maternal resources for that. In this study, health care decision-making autonomy which reflected the involvement of mothers in household decisions about their own health care, seeing a doctor and care of children during sickness was the most influential dimension on child growth. Health care autonomy perhaps allows mothers to make decisions in favour of children, and more likely to spend on health and nutrition^(43,44)^. The pathway that links low women's autonomy and child malnutrition is either through maternal own nutritional status which may affect the effective use of maternal resources or through reduced access to and control of household resources for childcare^(45)^.

There were other covariates for both the HAZ and stunted growth as presented in Tables 8 and 9. This included religion, gender of child, height and BMI of mother, bottle feeding practices and whether or not the child was wasted or not.

Relative to female children, male children had a higher probability of becoming stunted, a finding that has been reported by earlier studies^(46–48)^. The exact factors contributing to this male vulnerability is unclear but it is unlikely to be the result of gender preference^(46,49)^. The male vulnerability to undernutrition may be biological and the fact that male children are at greater risk of infection because of a greater tendency to explore the environment compared to female counterparts. It has been suggested that despite the improvement in medical care, environmental stresses have harsher effects on males than females in early life^(50)^.

Compared to children of ATR, Christians and Muslim children had a higher probability of being stunted. This is consistent with findings of other studies^(33)^.

Bottle-fed children had greater odds of becoming stunted, compared to their counterparts who were not bottle-fed. This may be because of increased infection associated with feeding bottles among women who for one reason or the other are unable to thoroughly clean these bottles. Interestingly, most of the other WHO recommended complementary feeding indicators (minimum meal frequency, minimum dietary diversity and MAD) were not associated with child growth indicators among children aged 6–23 months. The apparent lack of association may be due to the fact that there was very little variation in the study population with respect to these indicators. The lack of association may also be explained partly by the fact that the feeding indicators may not be sensitive to chronic undernutrition because they are assessed based on 24-hour recall which may not give the usual dietary intake.

Surprisingly, children who were not wasted had a greater likelihood of becoming stunted compared to children who were wasted. However, some studies including a recent one in the Gambia reported that being wasted was predictive of stunting^(51,52)^. The present study was cross-sectional and so though wasting could lead to stunting, that does not mean that all wasted children are stunted and non-wasted can equally be stunted at any given point in time. The finding also suggests that linear growth may take place in the presence of wasting, such that wasted children may not necessarily be stunted after all.

The results also show that a unit increase in maternal height was associated with 5 % protection against child stunting, and similarly, a unit increase in a mother's BMI was associated with 9 % protection against child stunting. This confirms earlier research findings that high maternal height^(53,54)^ is positively associated with improved child nutrition. The significant association between stunting and maternal characteristics indicates that nutrition interventions must equally focus on children and their mothers. It is indeed advocated that empowering women by improving their health is one of the best approaches to promoting the health and well-being of children in developing countries^(55)^.

The set of variables measured could accounted for 17⋅1 % of the variability in stunted growth in children. This is an indication that other factors contribute to the dependent variable but were not measured in this study.

### Association between women's autonomy and child feeding practices

There is a dearth of knowledge on the relationship between women's autonomy and IYCF, particularly with regards to which of dimensions of women's autonomy is relevant in addressing poor infant and child feeding practices. The present study investigated the association between WHO recommended IYCF practices and overall women's autonomy and its dimensions and the key finding was that overall maternal autonomy was associated with child feeding practices after controlling for key confounders such as age of child, bottle and breast-feeding status of child. IYCF practices are a major component of child caring practices.

The finding is consistent with previous studies^(17,36,56,57)^, which reported that maternal autonomy positively associated with child feeding practices especially improved dietary diversity in Vietnam, Ghana, South Asia and Sub-Saharan Africa. Similarly, several other studies have reported positive associations between women's empowerment and dietary diversity of the child^(58–60)^. When mothers have decision-making autonomy over child feeding and childcare, they may be more likely to follow recommended feeding practices or provide more appropriate care^(32)^.

An assessment of which dimensions of women's autonomy are associated with appropriate infant and child feeding practices was also made. The only component that associated positively with MAD in this study population was maternal financial independence. Similar results were obtained in a study involving ten African countries where higher women's economic empowerment was associated with a higher likelihood of feeding an MAD^(61)^.

The positive association between higher women's financial independence and appropriate child feeding practices is expected because access to diversified diets increases with economic empowerment. Indeed, the financial constraint has been reported to be one of the major reasons mothers are unable to feed children more meals with nutritious foods in many countries, including India, Ethiopia and Uganda mothers^(5,62,63)^. It has been reported also that higher power over the financial resources may translate to a higher likelihood of better resources allocated for child nutrition^(64)^.

In an analysis conducted using data from ten Demographic and Health Surveys, women's empowerment which involves the ability to influence decision-making in many life aspects found varied results in the countries. Whereas women's empowerment consistently and positively associated with multiple IYCF practices in Mali, Rwanda and Sierra Leone, there were null or mixed results in the remaining countries^(61)^. The study further concluded that women's empowerment for IYCF practices needs to be seen in terms of context and dimension of empowerment.

It has been reported that only one in three or one in six 6–23-month-old children in Sub-Saharan Africa were fed adequately diverse or overall acceptable diets, respectively^(65)^. Since women's autonomy has been shown to positively impact on feeding practices, it stands to reason that it should be aggressively be promoted towards optimal child care behaviours and better nutritional outcomes.

## Strengths and limitations of this study

The present study adds to the past work on the relationship between women's autonomy and child growth in a number of ways. First, it has collaborated past research showing a positive link between women's autonomy and child nutrition and also adds that the protective effect of autonomy is dependent on child's age. Though autonomy is a multidimensional construct, the majority of past research focuses on one of two dimensions of autonomy such as decision-making autonomy. This study differs in that several dimensions of maternal autonomy were measured concurrently as a multidimensional concept. The results have shown that different dimensions do have differing impacts on child nutrition and this is relevant to public health interventions because the most important aspects of women's autonomy were made clearer.

Furthermore, the study tested the relationship between women's autonomy and anthropometric measures of child growth and found that it associated with only length-for-age *Z*-score (LAZ) but not weight-for-age (WAZ) and weight-for-length (WLZ).

However, there are some limitations to the study which need to be considered. The analytical cross-sectional study design limits the ability to draw any causal conclusions since the problem of bias cannot be ruled out. Furthermore, one round of 24-hour dietary recall was used to calculate dietary intake. The method is retrospective and therefore depends on memory and the ability of respondents to recall accurately. Recall bias could not be ruled out completely. The 24-hour dietary recall may not truly represent the usual intake. However, a single 24 hour provides an estimate of mean intake of foods and nutrients^(66)^.

Another limitation of this study was that only women were interviewed. Ghuman *et al*.^(67)^ suggested that measurements of women's autonomy change depending on whom – a woman or her husband – was interviewed. Nonetheless, our results have thrown more light on the association between maternal autonomy and nutritional status of children less than 2 years in the Bawku West District of Ghana.

## Conclusions and recommendations

The findings of the study suggest that high women's autonomy was strongly and positively associated with mean height-for-age *Z*-score (HAZ and MAD). Therefore, policies and nutrition interventions focusing on women's autonomy may enhance women's ability to make health care decisions for themselves and their children, thereby speeding progress towards accomplishing many of the SDGs targeted at women and children.
